# Update in palliative management of hormone refractory cancer of prostate

**DOI:** 10.4103/0970-1591.30266

**Published:** 2007

**Authors:** Pratipal Singh, Aneesh Srivastava

**Affiliations:** Department of Urology, Sanjay Gandhi Post Graduate Institute of Medical Sciences, Lucknow, India

**Keywords:** Cancer prostate, hormone refractory, hormone refractory cancer of prostate, palliative care, prostate

## Abstract

Hormone refractory prostate cancer (HRPC) is an incurable disease and as in the pressure sensitive adhesive era the median survival of patients is increasing, these men increasingly develop symptomatic problems as a result of advanced local and or metastatic disease during their progression to death. Recently, it has been shown that it is possible to improve survival in this group of patients with use of chemotherapy which reinforces the need of better options in palliative care. We discus the various clinical problems (Part I) and treatment options of palliative care (Part II) and try to formulate an action plan in this review.

## CLINICAL PROBLEMS IN HORMONE REFRACTORY CANCER OF PROSTATE

Hormone refractory prostate cancer (HRPC) is amongst the most common and challenging in urological practice. Since the observations of Huggins and Hodges[1] in 1941, chemical or surgical castration has become the mainstay for the treatment of metastatic prostate cancer. Historically, all these patients on hormone therapy develop hormone resistance after an average of 18 to 24 months[2] and their prognosis has been stated to be dismal with a median survival of 12 to 18 months.[3] However; in a recent study median survival of HRPC patients with and without skeletal metastasis was 40 and 68 months, respectively.[4] It is defined as androgen insensitive prostate cancer (AIPC) when there is measurable progression of disease despite castrate serum testosterone levels as evidenced by at least one new lesion on bone scan or increasing pressure sensitive adhesive (PSA) (minimum 5 ng/ml with two consecutive increases of 50%), which is resistant to castration but sensitive to secondary hormonal manipulations and as hormone refractory prostate cancer when it is resistant to all hormonal manipulations.[5] Therefore, before labeling prostate cancer as an HRPC, ideally all secondary hormonal manipulations must be attempted, at least androgen withdrawal.

Hormone refractory prostate cancer is an incurable disease and as in the PSA era median survival of patients is increasing, these men increasingly develop symptomatic problems as a result of advanced local and or metastatic disease during their progression to death. These problems can be grouped as:

- Lower urinary tract problems
- Ureteric obstruction
- Skeletal problems
- Hematological problems
- Lymphoedema
- Rectal obstruction/infiltration
- Pelvic pain
- Neurological problems
- Psychological dysfunction/impaired quality of life

### Lower urinary tract problems

It is a common problem, however there are no good data to establish the true incidence of lower urinary tract dysfunction in men with end-stage prostate cancer. Main lower urinary tract problems are bladder outlet obstruction (BOO), urinary incontinence[6] and hematuria.[7] These problems arise due to encroachment of prostate cancer on the prostatic urethra and infiltration of the cancer into the bladder neck, distal sphincter and pelvic floor. This leads to bladder outflow obstruction and impaired urinary control, both of which may occur concurrently.

Urinary incontinence may be due to overflow as a result of BOO or due to damage to sphincter mechanism either by tumor infiltration or injudicious channel TURP. It is a distressing problem and can be a major constraint on normal social and family life.[6]

Hematuria can be a major source of physical and psychological morbidity and it is a frightening complication for patients. It is usually due to friable tumour invading the prostatic urethra. Platelet dysfunction and low-grade disseminated intravascular coagulation can also coexist in this group of patients which may exacerbate the problem.[7]

### Ureteric obstruction

This complication occurs in 2-3% of patients with HRPC[8] although its incidence is higher in some reports.[9] This can occur by direct extension of carcinoma into the trigone and bladder or from extrinsic pressure on the ureter by retroperitoneal lymph node metastasis, however, metastasis to the wall of the ureter or to the peri-ureteral lymphatics is less common. It can be unilateral or bilateral and may be clinically silent in early stages. It can present as flank pain, anuria, acute renal failure, sepsis or as biochemical disturbances.

### Skeletal problems

Skeletal metastases are found in 70% of patients with advanced prostate cancer and in more than 90% of patients who die of prostate cancer.[10] Clinical consequences can be bone pain, pathological fracture, spinal cord compression, hypercalcemia and hypocalcemia. Skeletal complications have been shown to significantly reduce health-related quality of life in patients with prostate cancer.[11]

#### Bone pain:

Many patients with HRPC will have bone pain at some point in the course of their disease.[12] Its cause is not completely understood, however, it is suspected that cancer-induced osteolysis may be involved. Bone pain has an inflammatory and a neuropathic component but it also appears to have unique neurochemical features that differ from either component.[13] Pain can be diffuse or localized and it is described as an ache, burn or stabbing sensation that increases at night. The extent of disease in the skeleton does not necessarily correlate with the pain experienced[14] but whether the pain is associated with a single isolated metastasis or with a heavy skeletal load, it is often a source of considerable morbidity requiring multidisciplinary treatments.

#### Pathological fractures:

Incidence of pathologic fracture in the old literature is around 3%[15] but recently it has been reported to be as high as 33% in HRPC, with about two-thirds occurring in the vertebral column and the rest in long bones.[16] This risk is exacerbated by the long-term use of androgen suppression[17] and when fracture occurs, it is associated with a significant reduction in patient survival.[18] Fracture can be diagnosed by plain X-rays, computed tomography (CT) or magnetic resonance imaging (MRI) once at least 30-50% destruction of cortical bone has taken place in any plane.

#### Spinal cord compression:

Prostate cancer is second only to lung cancer as a cause of metastatic spinal cord compression in men and 1-12% of prostate cancer patients will develop this complication.[19] In 12-19% of cases spinal cord compression is the first presenting sign.[19] It is possible to predict at risk population and extent of disease on bone scan and the duration of continuous hormone therapy are independent predictors of subarachnoid space or spinal cord (SAS/SC) compression.[20] The risk of occult SAS/SC compression increased from 32% to 44% in patients with more than 20 metastases on bone scan as the duration of hormone therapy increased from 0 to 24 months but the presence or absence of back pain was not predictive of SAS/ SC compression. The MRI in such patients helped to identify occult disease which was treated locally with radiotherapy before developing irreversible neurological signs.[20] There is also evidence to suggest that adding a bisphosphonate to the treatment of this group of patients will decrease the risk of decompression still further.[15] Spinal cord compression most commonly occurs in the thoracic and upper lumbar spine where the spinal canal is the narrowest[20] and results from either vertebral collapse after tumor invasion of vertebral body or by extradural tumor growth anywhere along the spinal canal.[21] The most common symptom is local or radicular pain. Paraparesis may develop which can be indolent and slowly progressive or acute and progress rapidly to paraplegia. Sensory deficits are frequently identifiable and autonomic dysfunction like hypotonic bladder may also develop.

Magnetic resonance imaging is now the radiological procedure of choice in the diagnosis and localization of spinal cord compression.[22] The sensitivity and specificity of MRI in the diagnosis of spinal cord compression due to extradural masses are similar to those of myelography.[23] Furthermore, MRI was more sensitive for diagnosing intramedullary metastasis, paravertebral masses and bony metastasis. Myelography is now reserved for patients with claustrophobia, pacemakers and metallic implants near the area of interest.[22]

#### Hypercalcemia and hypocalcemia:

Hypercalcemia is reportedly rare in prostate cancer.[24] Certain reasons may explain the low rate of symptomatic hypercalcemia in prostate cancer, including osteoblastic activation, which hampers massive calcium release into the bloodstream from the metastatic sites and induces a ‘calcium sink’ effect[15] and the possible role of PSA in inactivating parathyroid hormone-related protein produced by cancer cells.[25] In contrast, hypocalcemia is a metabolic disturbance more common in advanced prostate cancer due to the bone hunger syndrome. In a recent series hypercalcemia and hypocalcemia were seen in <1% patients, respectively but bone hunger syndrome was present in 13.4 % patients.[26]

### Hematological problems

#### Anemia:

It is a common finding in HRPC patients, however, true incidence is not known. It can be due to direct consequences of red marrow infiltration by metastatic prostate cancer cells, iron deficiency due to poor dietary intake or anemia of chronic malignancy and chronic disease. Anemia of prostate cancer develops gradually, therefore even profound anemia can be tolerated well. Lethargy and shortness of breath are common symptoms.

#### Coagulopathy:

Patients of carcinoma prostate can present with bleeding abnormalities and severity of bleeding may range from easily controlled hematuria to syndrome of disseminated intravascular coagulation (DIC).[7] Approximately 13% patients of carcinoma prostate develop some form of chronic DIC syndrome. The DIC in prostate cancer is most commonly of the chronic type and presents as low-grade bleeding tendency with or without venous thrombosis. Bleeding manifestations are usually mild and frequently intermittent over a period of many months. Diagnosis is made by assessing platelet counts, plasma fibrinogen level, fibrin levels, prothrombin time and partial thromboplastin time. Factor VIII level measurement may be required in a few cases.

#### Lymphoedema:

As prostate cancer spreads, it may obstruct lymphatic and venous outflow from the lower extremities and pelvis, resulting in lower extremity edema. It is rarely due to hypoalbuminemia. If the edema is unilateral, possibility of deep vein thrombosis should be considered and needs to be ruled out by appropriate investigations.

#### Rectal obstruction/infiltration:

Direct invasion of the rectum or involvement of perirectal areas by prostate cancer is common. Clinical manifestations are variable, commonest is constipation; others are rectal pain, tenesmus, rectal bleeding and in most severe form total bowel obstruction. Rectoprostatic fistula is quite an uncommon complication. Autopsy studies have indicated that rectal involvement by prostatic adenocarcinoma occurs in 4% of patients (range1-12%).[27] The rectal infiltration took the form of an anterior rectal mass with or without ulceration in 52%, an annular stricture in 45% and separate metastasis in 3%. In 40% of patients, a preceding history of prostatic adenocarcinoma was elicited at the time of gastrointestinal presentation, while in 60% it was not elicited.[27] Diagnosis is usually straightforward but at times it may be difficult to differentiate metastatic prostate cancer from primary adenocarcinoma of colon. In this situation clinical indicators of rectal obstruction secondary to prostatic carcinoma are: unilateral or bilateral ureteral obstruction, mucosal integrity over the obstructing lesion, characteristic osteoblastic bony metastasis, elevated acid phosphatase (prostatic fraction) and serum PSA levels.

#### Pelvic pain:

Locally advanced prostate cancer involving the rectum and sacral plexus can cause severe perineal pain of neuropathic origin or due to rectal involvement. Pain can also be due to bladder invasion or bony erosion.

#### Neurological problems:

Brain metastasis usually represents the failure of hormone deprivation therapy and the presence of disseminated disease. Metastasis to the brain can occur by way of Batson's plexus or by direct extension from adjacent structures such as the sphenoid bone or sinuses.[28] The most common intracranial sites of prostate cancer metastasis are the leptomeninges (67%), cerebrum (25 %) and cerebellum (8%).[29]

These metastases are usually clinically silent and rarely may present with nerve palsies or with signs of raised intracranial tension. Contrast enhanced MRI is the diagnostic modality of choice. Compared with CT scanning, MRI is more sensitive in detecting multiple metastases, especially at the gray white junction.[30]

### Psychological dysfunction/impaired quality of life

Psychological dysfunction is common in HRPC patients and up to 25% of patients with cancer develop clinical depression, which might potentially benefit from treatment.[31] In HRPC, it has been shown that depression occurs relatively early in the time course of the disease and it is present one year before death in many individuals.[32] It has also been shown recently that patients with better baseline HRQL have better predicted survival, time to disease progression and pain prognosis than those with worse HRQL.[33]

## PALLIATIVE CARE IN HORMONE REFRACTORY CANCER OF PROSTATE

Hormone refractory prostate cancer is an incurable disease but treatment options are available which will allow a comfortable death to the patient. Recently, it has been shown that it is possible to improve survival in this group of patients with use of chemotherapy.[34] Terms palliative and supportive care are used interchangeably and they include decreasing the physical, emotional and spiritual distress of patients or their family. It should not be equated with end of the care but it is integrated throughout the illness even when cure is not possible. In this section we discuss various treatment options of palliative care.

### Bladder outlet obstruction:

Acute onset obstruction requires indwelling urethral catheter or suprapubic cystostomy. Treatment of chronic BOO in patients failed on hormone therapy is transurethral prostatectomy but the surgical approach is modified limiting the resection to a ‘channel’. It is important to remember that normal anatomic landmarks may be obscured, anatomic plane between the capsule and prostate tissue is poorly defined, prostatic fossa and bladder neck may be rigidly fixed and there may be preexisting distal sphincter compromise due to tumor infiltration. The operation can be very effective in restoring voiding[35] although the incidence of urinary incontinence afterward is higher than for a conventional TURP[35] and the subsequent mortality is increased considerably over the TURP for benign disease.[36] However, in a recent study on patients with locally advanced prostate carcinoma, after channel TURP initial voiding trial failed in 42, 29% required repeat procedure for bleeding or obstruction and 21% ultimately required long-term bladder drainage.[37]

Urethral stents are another option for obstruction if the patient cannot tolerate the transurethral resection procedure and refuses long-term catheter placement. After stent placement 88-100% of patients are able to void. This is best considered in patients with limited life expectancy due to possible tumor growth into the stent, causing obstruction.

### Hematuria:

Bleeding usually settles with conservative measures such as stopping drugs with an antiplatelet action, optimization or cessation of anticoagulants and bladder irrigation where necessary.[8] Inflating the balloon of Foley catheter with 30cc and mild traction on it may help to tamponade bleeding but it should not be applied for more than 24h as it may result in ischemic damage and secondary bleeding from the urothelium. Endoscopic resection and or fulguration for hematuria is indicated with failure to clear clots with hand irrigation, a persistently falling hematocrit or inability to significantly clear urine after 24h. In refractory cases trial of 0.5 to 1% alum irrigation should be given. This is generally well tolerated and safe although aluminium toxicity can be a potential problem if there are large vascular channels opened in a patient with compromised renal function.

Local radiotherapy is effective in controlling hematuria in locally advanced prostate cancer[38] and palliative treatment using this method is worthwhile in refractory cases not previously treated in this way. However, it will not usually help in alleviating the irritative or obstructive outlet symptoms.[39]

### Urinary incontinence:

It is usually best managed by catheterization. A urethral indwelling catheter is often useful although suprapubic catheters may be less problematic. However, these are not effective when the sphincter mechanisms have been damaged irreparably either by tumor infiltration or due to injudicious TURP and condom drainage remains the only answer.[6] Urinary diversion is rarely indicated in these patients except where there is a rectoprostatic fistula.

### Management of ureteric obstruction:

Several modes of therapy are available to treat it effectively but the treatment depends on various factors, including the nature and severity of the obstruction, general condition of the patient, extent of the disease and perhaps most importantly, patient's wishes as median time from nephrostomy drainage to death in men with HRPC can be as little as three months, with or without nephrostomy drainage, if no other treatment option is available.[40]

Cystoscopy with retrograde placement of ureteral stents can be attempted but is usually not possible due to significant obstruction. Percutaneous nephrostomy (PCN) tubes may be easily placed under ultrasonic or C-arm guidance and can provide immediate relief of obstruction. An indwelling ureteral stent can replace the external nephrostomy by antegrade placement through the nephrostomy site. Placing and exchanging stents may be difficult if tumor has invaded the distal ureter and trigone.

Steroids can be used to obtain short-term decompression[41] and radiotherapy to nodal masses or bladder base also has response in up to 70% cases[42] but effects are evident in four to five weeks and this time needs to be bridged by ureteric stent or a PCN.

### Skeletal problems

#### Bone pain:

Treatment options include radiotherapy for bone lesions, decreasing tumor-induced bone loss, surgical stabilization of osteolytic weight-bearing bones and intensive use of narcotic analgesics (opioids).

Focal external beam radiation is effective with 90% of patients experiencing some pain relief and 54% experiencing a period of complete relief when pain is localized to one site or one area, however, 50% of patients who have some pain relief return to pretreatment levels.[43] Further radiotherapy is possible if pain recurs. Symptomatic focal metastasis should be treated with focal external beam radiation to limit tissue damage and minimize anemia. If long bones are involved, the full length must be treated to prevent tumor recurrence in the marrow and at intramedullary sites. Weight-bearing bones with greater than 50% of the cortex eroded on plain X-ray or computerized tomography require surgical stabilization prior to treatment. The use of ‘single-shot’ radiotherapy seems to be as effective as multi-fraction treatment in this situation (single-fraction 8 Gy versus 10 fractions of 3 Gy) with no significant difference in outcome.[44]

When many sites are painful or where there is a typical ‘flitting’ bone pain, hemi-body irradiation (HBI) or the administration of systemic radionuclides is effective. Hemi-body irradiation will induce responses in 91% of patients (45% complete responses) within three to eight days and fractionated treatment is most effective in this setting.[45] Systemic radiotherapy using bone-seeking isotopes can be very effective in multiple site metastases. The most commonly used agent is ^89^strontium, although ^32^phosphorus, ^155^samarium and ^86^rhenium are also effective. ^89^Strontium is a calcium analogue that is deposited by osteoblasts near metastatic tumors. Shallow-penetrating, low-energy particles are emitted with minimal damage to surrounding tissue. Initially pain may increase but eventually a decrease is seen in 80% of patients with 10% showing complete relief for three to six months.[46] Care must be taken to rule out metastatic masses that may become edematous and compress the spinal cord after treatment. Anemia, pancytopenia and pneumonitis are other possible side-effects. Its overall efficacy compares favorably with HBI[47] and it is less toxic; its main drawback is its high cost.

The role of bisphosphonates in alleviating bone pain is uncertain. Initial and less potent first and second generation bisphosphonates have shown some beneficial effects only in open label studies but current third generation bisphosphonates are much potent and have shown good results in randomized trials. In a large-scale randomized trial using zoledronic acid, currently the most potent bisphosphonate available, there was a significant decrease in pain and requirement of analgesics for those receiving the drug, although the quality of life and performance were not affected significantly.[48] It seems that the patients most likely to benefit from this class of drugs as a pain-relieving therapy are those with painful bone metastases which are difficult to control by other means.

Chemotherapy has been disappointing with response rates far less than those of radiation treatment for bone pain. Mitoxantrone in combination with prednisolone has shown pain relief in 29% patients, however, docetaxel with prednisolone has given superior results than mitoxantrone and prednisolone and pain relief was seen in up to 35% patients of HRPC.[34]

Glucocorticoids can also decrease bone pain. The mechanism is unclear but inhibiting prostaglandin release may prevent the hypersensitization of peripheral nerves and decrease edema near lesions to decrease pressure. Oral prednisone (20 to 40mg daily) is effective but even lower equivalent doses have been used. The pain decrease is usually short-lived and so glucocorticoids should be used as a bridge between more long-acting treatments. Glucocorticoids and nonsteroidal anti-inflammatory drugs (NSAIDs) should not be used together because toxicity is increased without any significant increase in the analgesic effect.

Analgesic treatment should not begin until a reliable assessment of pain severity is completed for mild pain and it should begin with oral acetaminophen or NSAIDs.[49] Opioids are the mainstay for moderate to severe pain and they can be used in combination with NSAIDS or acetaminophen. Short-acting morphine is often used initially and once pain is under control, a long-acting form with limited supply of short-acting forms for breakthrough pain is appropriate. Overdose is rarely a problem, however, under-treatment of pain is a frequent quality of care issue. The dose should be increased until pain is controlled or when adverse effects become intolerable. There is no maximum dose for most opioids but care must be taken with opioids containing acetaminophen to avoid dosing beyond the maximum daily dose of, 4000 mg daily. Constipation is the most common complaint therefore stool softeners as well as bulk laxatives should be used. Most importantly the clinician can be successful by accurately assessing and then reassessing pain severity after intervention. This approach to pain management is a consensus validated approach to pain relief.[49]

#### Pathological fractures:

Pathologic fractures can have especially grim implications because the majority requires radiotherapy and/or orthopedic surgery. Additionally, extensive rehabilitation is required to restore function when weight-bearing bones are involved.[50] Surgery for pathologic fracture is associated with a 4% postoperative fatality rate.[51] Furthermore, surgery fails to restore mobility in approximately 25% of patients with pathologic fractures of the long bones of the leg.[52] An emerging body of evidence now suggests that the incidence of fracture can be reduced by using bisphosphonates. Current treatment algorithms for patients with prostate cancer support the use of zoledronic acid to reduce the incidence of skeletal complications from bone metastases and to prevent bone loss during ADT.[53] The patients who are most likely to benefit from routine use of bisphosphonates with calcium and vitamin D supplementation are those with a heavy skeletal tumour load, particularly in the thoracolumbar spine and femoral bones and those who have been on long-term LHRH therapy or maximal androgen blockade.

Further prophylactic treatment measures include local irradiation of lytic metastases in the shafts of long bones and in the neck of the femur. However, when a pathological fracture occurs in a long bone, it is important to treat this by rapid orthopedic fixation or joint replacement which is very effective in reducing pain and restoring mobility. Fixation is usually supplemented by treatment with local radiotherapy after two to four weeks of the surgery. Life expectancy should not usually affect the decision for or against surgery, nor should the inability to ambulate prior to the presentation of the metastasis; as stabilization may make bed-to-chair transfers painless and facilitate terminal nursing care.[50]

#### Spinal cord compression:

The goal in the treatment of spinal cord compression secondary to metastatic prostate carcinoma is palliation. Pain relief and prevention of neurologic deterioration through local tumor control are of primary importance. Steroids should be administered immediately in order to help reduce edema around the spinal cord. A standard protocol is 100 mg dexamethasone administered intravenously, followed by 24 mg every six hours which can be tapered after two days. Ultimately, the patient may be started on oral corticosteroids once his clinical condition permits.

There is no clear advantage for either radiation or surgery as the next step in the definitive therapy of spinal cord compression.[54,55] Most studies have concluded that there is no distinct advantage of one therapeutic approach over the other, although both modalities can provide rapid pain relief in nearly two-thirds of patients. Immediate surgical decompression should be used in patients with an expected lifespan of at least six months, who deteriorate during radiation, who have had previous radiation in the involved site, who have a potentially correctable unstable spine or who had spine compression of unknown histology.[56]

It is widely accepted that the neurologic status prior to treatment is a major determinant influencing the outcome as in most series 100% patients who are ambulatory prior to treatment, more than 50% patients with paraparesis and less than 35% patients with paraplegia become ambulatory after treatment. The younger, more ambulant man, preferably with a single compression site, is the one most likely to benefit, provided that the decompression is undertaken as soon as possible after the onset of symptoms.

#### Hematological problems:

Anemia and coagulopathy are two important hematological problems associated with HRPC. Conservative treatment measures, such as iron and vitamin supplementation and the use of bone marrow stimulants, such as erythropoietin, may be helpful in some patients, although it is common for transfusion dependency to emerge as the effects of chronic disease and suboptimal bone marrow productivity take their toll. Therapy with transfusion for anemia depends primarily on the patient's symptoms as well as such factors as other illnesses and health status. There are no absolute indications for transfusion if the hemoglobin is above 6 to 7g/100 ml, but transfusion should be considered in a symptomatic patient.

A few studies examined the use of the erythropoietin in HRPC. In a small study,[57] four of nine patients receiving treatment over a 12-week period had a 20g/L increase in their hemoglobin levels. In another study, patients receiving erythropoietin at a dose of 5000 units three times/week benefited significantly with no major side-effects. The quality of life in the 43% of patients having an increase in hemoglobin of >20g/L was improved.[58] However, given the life expectancy of the patients and the cost of therapy, the treatment cost/benefit ratio must be considered and therefore, probably, erythropoietin therapy has not yet gained wide acceptance in the treatment of anemia arising from HRPC.

Treatment of DIC ideally would impact the causative factor, the prostate cancer itself. Given the difficulties in successful control of the cancer, treatments have often been shifted to managing the coagulopathy. Unfortunately, benefits from this approach have yet to be demonstrated conclusively. Nevertheless, some authors recommend intravenous heparin and epsilon aminocaproic acid (EACA) to treat DIC. There are, however, significant safety and efficacy considerations in using this combination of heparin and EACA. Heparin may cause a worsening of DIC and fatal hemorrhage. Additionally, EACA inhibits circulating plasminogen activators and the fibrinolytic system, possibly contributing to bleeding or thrombosis. At some point, patients with coagulation system dysfunction may need to be supplemented with specific elements (e.g, platelets, fresh whole blood, packed erythrocytes, frozen plasma or cryoprecipitate).

#### Lymphedema:

Treatment of lymphedema is based on physical maneuvers, pharmacological agents being of little benefit.[59] Measures to limit the problem include simple limb elevation in mild cases, gradual compression stockings in moderate cases and intermittent use of pneumatic compression pumps in severe cases.[6] In patients refractory to hormones, external irradiation is sometimes delivered to large pelvic masses that appear to compromise the lymphatic or venous outflow. However, this may compound the problem rather than relieve it.

#### Rectal obstruction/infiltration:

Local measures to alleviate the rectal obstruction include transanal resection[60] and local treatment with external beam radiotherapy. The latter is used more widely but its true utility has not been tested in a controlled manner. If constipation becomes severe and intractable or total obstruction of the bowel develops, diversion colostomy should be done, preferably as an elective surgery rather than as an emergency.[29] Rectoprostatic fistula is fortunately an uncommon occurrence but once it develops urinary and fecal diversion is the only way to obtain control of body effluent in a satisfactory palliative manner. In a recent study the median survival was 15 months (95% confidence interval 14-16 months) and survival beyond 30 months was rare in patients of HRPC with rectal involvement.[27]

#### Pelvic pain:

It is a difficult problem to treat in HRPC patients if analgesics have failed. It can be alleviated in appropriate cases by sacral nerve ablation using caudal injection of alcohol or phenol as a neurolytic. This is an effective way of eliminating pelvic pain, but it will also impair emptying functions of both the bladder and the rectum. Another alternative is regional analgesia using procedures usually performed by an anesthesia pain expert.

#### Neurological problems:

In patients with brain metastasis, unless seizures occur, the use of prophylactic anticonvulsants, particularly phenytoin, is not encouraged as in combination with radiotherapy, phenytoin may cause Stevens-Johnson syndrome.[61] Dexamethasone therapy should be started early. A two-week course of radiotherapy is the most common treatment for patients with multiple brain metastases or leptomeningeal involvement. Surgical removal of a solitary lesion may extend survival.[62]

#### Psychological dysfunction:

Urologists should be aware of this problem and in patients with sustained symptoms which are refractory to standard medication, due consideration should be given to referral for psychiatric counseling and the involvement of other support groups.[6]

## CONCLUSION

In the era of PSA and effective chemotherapy for HRPC leading to improved median survival, the need for patient-centered and focused palliative care cannot be overemphasized. It is very important to define the basic clinical problem and provide the appropriate care in a judicious manner.
